# Mycobacterium abscessus Complex Osteomyelitis of the Wrist

**DOI:** 10.7759/cureus.32593

**Published:** 2022-12-16

**Authors:** Noah Alter, Robert W Trenschel, Gary Schwartz, Joshua Sharan, Ryan P Boyle, Mohammadali M Shoja

**Affiliations:** 1 Department of Medical Education, Dr. Kiran C. Patel College of Allopathic Medicine (NSU MD) Nova Southeastern University (NSU), Fort Lauderdale, USA; 2 Department of Medical Education, Dr. Kiran C. Patel College of Osteopathic Medicine (NSU-KPCOM) Nova Southeastern University (NSU), Fort Lauderdale, USA

**Keywords:** osteomyelitis, mycobacterium, infection, bone, antibiotics

## Abstract

*Mycobacterium abscessus* (*M. abscessus*) is a ubiquitous, rapidly growing non-tuberculous mycobacterium, which is known to cause life-threatening lung infections in immunocompromised individuals following exposure to contaminated injectable products. We report a case of *M. abscessus* osteomyelitis of the right wrist in a 28-year-old patient with a history of intravenous drug use and a recent surgical repair of the right radial artery pseudoaneurysm. The patient underwent surgical debridement of the right distal radius infection. Histopathological examination and culture of the debrided tissue revealed *M. abscessus *complex infection. The patient was placed on intravenous amikacin, azithromycin, and cefoxitin for six weeks, followed by oral linezolid and clofazimine for six months.

## Introduction

*Mycobacterium abscessus (M. abscessus*) is a non-tuberculous mycobacterium that recently underwent a taxonomic overhaul with three separate subspecies being named: *M. abscessus* subsp. *abscessus*, subsp. *malliense*, and subsp. *bolletii*, all of which are rarely encountered clinically. *M. abscessus* is a ubiquitous organism found in water supply, soil, and dust [1]. As an opportunistic pathogen, it typically causes pulmonary, skin, and soft tissue infections [1,2,3,4]. It has the ability to cause cutaneous and soft tissue infections following trauma, as well as organ failure [5]. It is easily transmissible from human to human and is reportedly the most pathogenic rapid-growing mycobacterium. Because of its antibiotic resistance, *M. abscessus* is considered one of the most difficult rapidly growing mycobacterial infections to treat [6]. The difficulty in treating patients infected with this organism is made even more challenging by the lack of a standard treatment protocol for osteoarticular infections, although treatment guidelines do exist for pulmonary infections. We present the case of a patient with *M. abscessus* osteomyelitis of the wrist, who was treated by surgical debridement and prolonged systemic antibiotics.

## Case presentation

**Figure 1:**
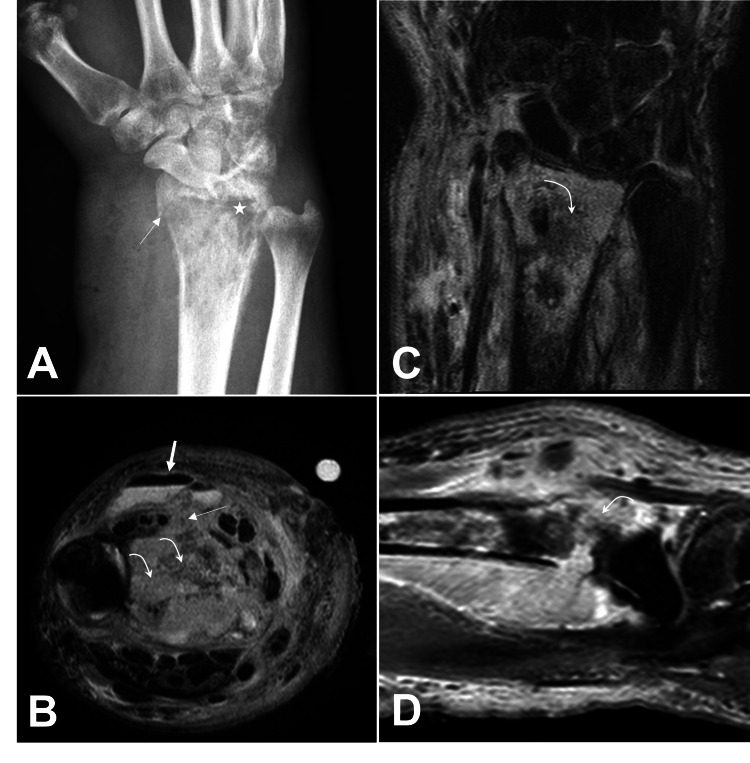
Preoperative imaging Preoperative anteroposterior view X-ray (A) of the right wrist demonstrates a permeative appearance and lytic lesions (star) of the distal radius along with a pathological fracture (thin arrow). Preoperative axial T2-weighted (B), coronal T2-weighted (C), and sagittal T1-weighted (D) MRI of the right wrist demonstrate evidence of osteomyelitis of the distal radius. There is a peripherally enhancing air-fluid collection (thick straight arrow) in the dorsal wrist with phlegmon extending into the distal radius (thin straight arrow). Note T2-weighted hyperintense marrow signal and enhancement within the distal radius (curved arrow) with cortical irregularity and periosteal reaction. Cortical disruption is noted volarly and dorsally MRI: magnetic resonance imaging

A 28-year-old male with a history of intravenous drug use presented to the emergency room with right wrist pain. He was HIV-negative. Two months earlier, he had been treated for discitis of T7-T8 with an epidural abscess, which had grown methicillin-sensitive *Staphylococcus aureus*. One month previously, he had undergone repair of the right radial artery pseudoaneurysm. The X-rays taken at the time of the pseudoaneurysm repair did not demonstrate any bony abnormalities. Additionally, on physical examination, the right wrist was tender, swollen, erythematous, and associated with a subcutaneous pus collection on the dorsal aspect, which was incised and drained at the bedside. The X-rays demonstrated severe soft tissue swelling, osteopenia, and a lytic lesion involving the right distal radius associated with a pathologic fracture (Figure 1A). An MRI demonstrated a pathologic right distal radius fracture with evidence of osteomyelitis (Figures 1B, 1C, 1D). The patient was initially placed on intravenous piperacillin/tazobactam, cefepime, and vancomycin.

**Figure 2:**
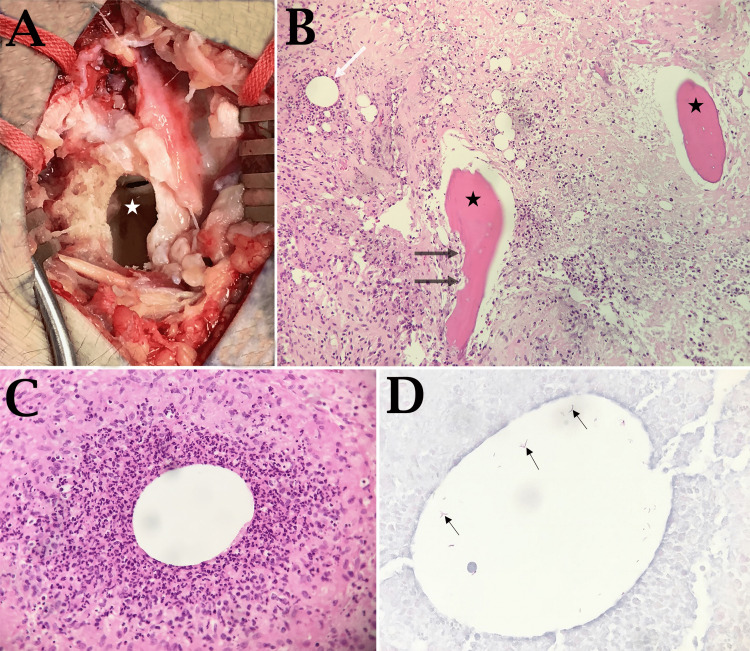
Intraoperative and histopathological findings (A) Intraoperative view of the dorsal right wrist demonstrating a large defect (star) in the distal radius. (B) H&E staining of the debrided distal radius specimen shows necrotic bone trabeculae (black star) with empty osteocyte cavities. Note the irregular trabecular contours being destroyed by the inflammatory cells (thick black arrows). There is intramedullary microabscess formation (white arrow). (C) H&E staining of the marrow space shows acute inflammation and microabscess formations. Note the epithelioid reaction and central vacuole surrounded by the neutrophilic infiltrate. (D) Acid-fast staining of the tissue shows acid-fast bacilli inside the vacuoles (thin black arrows)

The patient underwent extensive debridement of the right distal radius (Figure 2A). Histopathological examination of the right wrist synovium and distal radius showed acute and chronic osteomyelitis with necrotizing granulomatous inflammation, giant cell reaction, and the presence of acid-fast bacilli (Figures 2B, 2C, 2D). The acid-fast organisms were also Gram stain-positive and weakly Gomori methenamine silver (GMS) stain-positive. Many of the acid-fast organisms appeared clustered within cystic, lacunar-like spaces surrounded by neutrophils (Figure 2D). The tissue PCR testing was negative for non-tuberculous mycobacterium (*Mycobacterium avium* complex and rapidly growing mycobacteria) or *Mycobacterium tuberculosis* (*M. tuberculosis*) complex DNA (using 16s rDNA, hsp65, and rpoB primer sets). The bone culture grew *M. abscessus*, which was susceptible to amikacin, and intermediately susceptible to cefoxitin, minocycline, and imipenem but resistant to ciprofloxacin, clarithromycin, and doxycycline. The antibiotics were changed to intravenous amikacin, azithromycin, and cefoxitin for six weeks. He was then placed on oral linezolid and clofazimine for six months. On postoperative week six, the patient was found to have minimal discomfort and minimal swelling in the area of the right wrist, and the digital motion was excellent.

## Discussion

M. abscessus is a distantly related bacterium to *M. tuberculosis* and is known to cause life-threatening pulmonary infections in immunocompromised individuals in cases of exposure to contaminated injectable products [7,8]. It commonly occurs following a bacterial introduction via surgical wounds, environmental exposure, and/or traumatic injury. This organism is known to be highly resistant to many common antimicrobials. A combination of clarithromycin, amikacin, and cefoxitin has been used as the treatment of choice [3]. On culture, *M. abscessus* has demonstrated the least resistance to cefoxitin, azithromycin, and amikacin. The literature suggests that when treating *M. abscessus*, dual beta-lactams have synergistic benefits [9,10].

The wrist is a common location for non-tuberculous mycobacterial infection. Our patient was an intravenous drug user and had a recent history of right forearm surgery; it is suspected that his right wrist osteomyelitis occurred due to mycobacterial introduction following repeated injections using *M. abscessus*-laden recreational drug paraphernalia or by nosocomial means. Specifically, several studies in the literature have evaluated the transmission of infectious agents such as *Staphylococcus aureus* through injectables [11]. Analogously, we believe that our patient's* M. abscessus* infection occurred through a similar route.

There are scarce data in the literature regarding *M. abscessus* infections. Studies on *M. abscessus* osteomyelitis are even rarer. This pathogen is characterized as life-threatening in the geriatric community and among individuals with AIDS [3,12]. *M. abscessus* can also infect immunocompetent individuals. Kang et al. have reported *M. abscessus* infections in two immunocompetent fishermen following repeated minor traumatic injuries [7]. Similarly, in a systematic evaluation of patients from 2000 to 2015 in the Mayo Clinic’s Department of Orthopedic Surgery, Sotello et al. reported 15 immunocompetent patients who were infected with *M. abscessus* [13]. In these studies, *M. abscessus* strains had multidrug resistance. Early pathogen identification and implementation of an appropriate antibiotic regimen are essential for eradicating the organism [3].

Garcia et al. have reported a patient with a history of hepatitis C infection who was later diagnosed with *M. abscessus* osteomyelitis of the thoracic spine; the infection resolved with six-month treatment with cefoxitin, amikacin, and clarithromycin [14]. Damodar et al. have discussed a case of *M. bolletii *osteomyelitis of distal radius in a patient who had sustained an open fracture of the right distal radius one month earlier; the infection was cleared with debridement and placement of amikacin antibiotic beads in addition to systemic antibiotic therapy [15]. Gonzales Zamora and Villar Astete have reported a case of *M. abscessu*s osteomyelitis of distal phalanx in a patient one week after having a manicure; this was treated with six weeks of imipenem, tigecycline, and linezolid, followed by oral clarithromycin and linezolid for six months [16].

## Conclusions

*M. abscesses* osteomyelitis is a rare infection. A diagnosis of this condition is made by histopathological examination of the infected bone materials and culture. Characteristic histopathological findings are acute and chronic osteomyelitis with necrotizing granulomatous inflammation, epithelioid reaction microabscess formations and central vacuole surrounded by a neutrophilic infiltrate, and the presence of acid-fast bacilli. We implemented a combined medical and surgical approach for the treatment of *M. abscessus* osteomyelitis. A combination of clarithromycin, amikacin, and cefoxitin is a common antibiotic regimen for these patients, but the treatment should be tailored based on the antibiotic sensitivity results and may be extended for several months to eradicate the organism.
